# Computational modeling and synthesis of Pyridine variants of Benzoyl-Phenoxy-Acetamide with high glioblastoma cytotoxicity and brain tumor penetration

**DOI:** 10.21203/rs.3.rs-2773503/v1

**Published:** 2023-04-20

**Authors:** Charles Ingraham IV, Joanna Stalinska, Sean Carson, Susan Colley, Monika Rak, Adam Lassak, Krzysztof Reiss, Branko Jursic

**Affiliations:** Louisisan State University; Jagiellonian University; University of New Orleans; Louisisan State University; Louisisan State University; Louisisan State University; Louisisan State University; University of New Orleans

## Abstract

Glioblastomas are highly aggressive brain tumors for which therapeutic options are very limited. In a quest for new anti-glioblastoma drugs, we focused on specific structural modifications of benzoyl-phenoxy-acetamide (BPA) present in a common lipid-lowering drug, fenofibrate, and in our first prototype glioblastoma drug, PP1. Here, we propose extensive computational analyses to improve selection of the most effective glioblastoma drug candidates. Initially over 100 structural BPA variations were analyzed and their physicochemical properties such as water solubility (−logS), calculated partition coefficient (ClogP), probability for BBB crossing (BBB_SCORE), probability for CNS penetration (CNS-MPO) and calculated cardiotoxicity (hERG), were evaluated. This integrated approach allowed us to select pyridine variants of BPA that show improved BBB penetration, water solubility, and low cardiotoxicity. Herein the top 24 compounds were synthesized and analyzed in cell culture. Six of them demonstrated glioblastoma toxicity with IC50 ranging from 0.59 to 3.24mM. Importantly, one of the compounds, HR68, accumulated in the brain tumor tissue at 3.7+/−0.5mM, which exceeds its glioblastoma IC50 (1.17mM) by over 3-fold.

## Introduction

Glioblastomas are the most aggressive brain neoplasms with a dismally low 5 year patient survival rate of below 5% ^1^. According to the World Health Organization (WHO) glioblastomas are classified as grade I and grade II (low-grade gliomas), grade III (anaplastic), and grade IV (glioblastoma) ^2^. Current standard-of-care therapies include maximal surgical resection, followed by radiotherapy plus concomitant and maintenance temozolomide (TMZ) ^3^. In addition, a large variety of different genetic and epigenetic modifications have been found in glioblastomas, among which p53, EGFR, PTEN, and IDH mutations are the most common ^4–9^. However, these validated molecular targets, as well as immunotherapies, including immune checkpoint inhibitors ^10^, tumor vaccines ^11^, and chimeric antigen receptor T cell (CAR T) therapies ^12^ have all been extensively studied, but failed to significantly improve therapeutic outcome in glioblastoma patients.

There are several reasons why it is difficult to develop more effective glioblastoma therapy: (1) Glioblastomas are characterized by many dysregulated pathways that cannot be blocked simultaneously *via* a single therapy ^13^: (2) Glioblastomas are highly infiltrating and heterogenous tumors that are very difficult to remove by surgical resection without compromising function of surrounding brain areas ^14^; (3) It is difficult to diagnose glioblastoma in its early stages, therefore, large highly infiltrating and vascularized tumors are often present at diagnosis ^15^; (4) Use of rodent syngeneic and patient-derived models are common to optimize clinical protocols. One major problem is that these experimental tumors are typically ~ 10^3^-10^4^ smaller than human tumors, therefore data from drug delivery, drug retention, and tissue penetration experiments obtained from these small animal models are difficult to extrapolate to glioblastoma patients ^16^; and finally, (5) The blood brain barrier (BBB) prevents the majority of anticancer drugs from reaching tumor sites at clinically relevant concentrations, and current methods to enhance BBB penetration are not very effective for glioblastoma patients ^17^.

One drug that readily crosses the BBB is temozolomide (TMZ). Upon oral administration, TMZ maximum plasma concentration can be reached in about one hour, and the elimination half-life is approximately 1.8 hours. Importantly, penetration efficiency of TMZ into the central nervous system (CNS) is experimentally estimated to be about 20% of plasma levels. This is important because, applying this estimate to calculate logBB (Brain-Blood Distribution) ^18^ this equation produces a value of −0.7, which indicates a high capability of the compound to cross the BBB. In spite of these positive features, TMZ-treated glioblastoma patients develop TMZ-resistance and recurrent tumors are practically incurable ^19^. In addition, TMZ has been used in combination with other drugs, which enhanced its therapeutic effects. One example is a combination of TMZ with lipid lowering drugs, including statins ^20^ and fibrates, like for instance fenofibrate **(FF)**, which has strong anti-glioblastoma activity in cell culture, and in glioblastoma intracranial mouse models ^21^. However, we have also found, that **FF** ability to cross the BBB is low, and the compound is quickly processed by blood and tissue esterases to form PPARa agonist, fenofibric acid **(FFA)**, which is no longer effective in triggering tumor cell death ^22^. We made numerous modifications to the FF chemical structure and selected our first drug candidate, PP1 ^23^, which similar to FF blocks mitochondrial respiration and triggers a severe ATP depletion. With both substances, this is followed by phosphorylation/activation of AMP-activated protein kinase (AMPK - intracellular energy sensor), blockade of p70S6K phosphorylation (marker of active protein synthesis), activation of autophagy (p62 degradation), and extensive glioblastoma cell death ^24^. In spite of these promising results, and in spite of the fact that we detected PP1 in the brain at therapeutically relevant concentrations (Supplementary materials, page 248) PP1 anti-glioblastoma therapeutic effects were only marginal when the treatment was applied to large intracranial tumors ^24^. These data indicate that additional adjustments to the BPA scaffold, focused on improving compound cytotoxicity, BBB penetration and retention in the brain tumor tissue, are continuously needed.

## Results And Discussion

In early stages of drug design it is important to evaluate relevant physicochemical properties of prospective drug candidates. For over two decades, Lipinski’s rule of five was the gold standard in drug design ^25^. For drugs associated with the CNS, their ability to cross the brain blood barrier (BBB) is the most important characteristic and is not directly addressed by the Lipinski’s rule of five. Recently, two computational scores have been introduced to evaluate the probability of new drug candidates to penetrate the CNS. One is CNS Multiparameter Optimization (CNS-MPO) algorithm ^26^, which uses 6 physicochemical properties [ClogP (calculated partition coefficient - lipophilicity), ClogD (calculated distribution coefficient at physiological pH (7.4) – lipophilicity), MW (molecular weight), TPSA (topological polar surface area), HBD (hydrogen bond donor at pH = 7), and pKa (−log acid dissociation constant)] for estimating the probability of entering the CNS. The values for CNS-MPO score range between 0 and 6, and the values ≥ 4.0 have been used as cut-offs for compounds with increased ability to penetrate the CNS ^26^. Another is the Blood-Brain Barrier Score (BBB_SCORE) ^27^. BBB_SCORE is based on five physicochemical including number of aromatic rings, heavy atoms, MWHBN (a value comprising molecular weight, hydrogen bond donor, and hydrogen bond acceptor), pKa and topological polar surface area. Similar to CNS-MPO, BBB_SCORE also considers 4.0 as the cut-off for the acceptable BBB penetration ^27^. Consequently, the CNS-MPO and BBB_SCORE serve as complementary algorithms in which CNS-MPO provides information regarding probability of the compound to be found in the CNS, and BBB_SCORE is oriented towards specific physiochemical properties that increase probability of the compound to cross the BBB.

In regard to toxicity, one of the most common hurdle in drug testing is cardiotoxicity triggered by inhibition of cardiac potassium channel coded by the human ether-à-go-go-related gene (hERG). This toxicity test became a mandatory requirement for drug design and development, and can be calculated by the hERG algorithm ^28 29^. Based on the compound structure, hERG algorithm gives a score which is indictive of the compound’s inhibitory effect towards the hERG. In other words, for the drug to be considered safe, it’s hERG IC50 should be significantly higher than its therapeutic IC50 ^28^.

**Figure 1:**
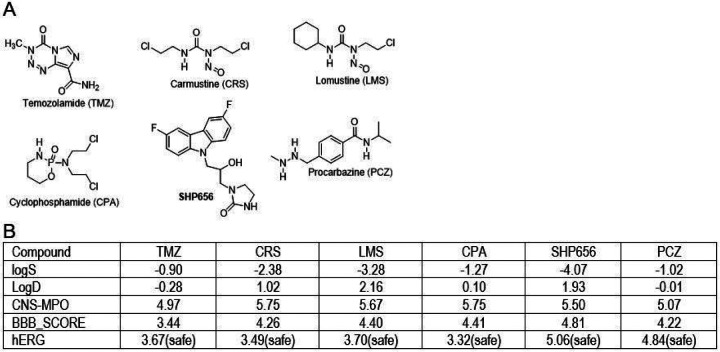
**Panel A**: *Chemical structures of the most common chemotherapy drugs used to treat brain and spinal cord and their computed psychopharmacological properties *. ***Panel B:****Physicochemical properties calculated for the compounds depicted in Panel A. LogS = water solubility; logD = distribution at pH 7.4; CNS-MPO = CNS multiparameter optimization algorithm; BBB_SCORE = blood-brain barrier penetration score; hERG = estimated pIC50 value for hERG (the human ether-a-go-go (hERG) potassium channel)*.

In order to determine glioblastoma-specific and therapeutically relevant values for all proposed algorithms/scores, including hERG, we have selected fifteen drugs (see Supplementary Materials, pages 57–79) that are either currently used as glioblastoma drugs, or are in clinical trials for glioblastoma patients ^30^. Results in Figure 1B show that six of these compounds (Figure 1A) have relatively high water solubility (LogS<0), low lipophilicity (logD<3), acceptable values for CNS-MPO and BBB_SCORE (ranging between 3 and 5), and low cardiac toxicity (hERG£5.5). We used these critical values as a guideline for designing new anti-glioblastoma drug candidates, which are based on the BPA chemical structure present in a common lipid lowering drug, fenofibrate ^21,22,31,32^, and in our first glioblastoma prototype drug, PP1^23,24^.

**Figure 2:**
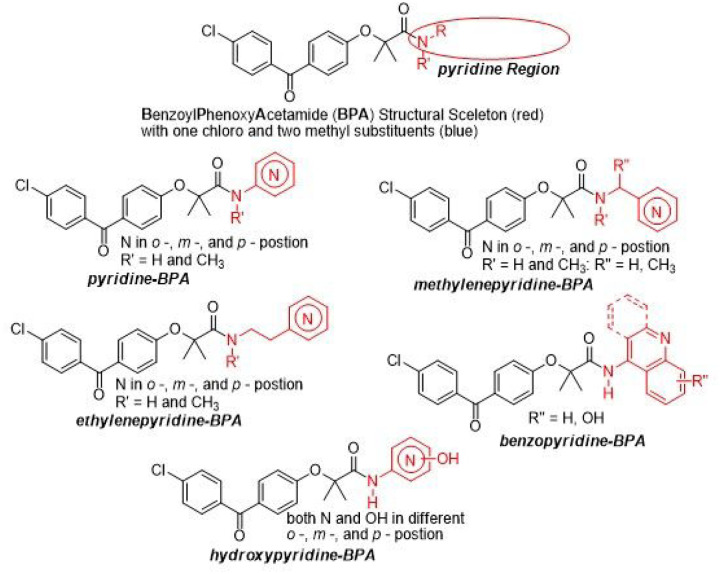
The pyridine region of BPA skeleton was selected for modification (circle) in search of the optimal anti-glioblastoma drug.

In search for more effective benzoyl-phenoxy-acetamide (**BPA**) derivatives, we have decided to explore pyridine variants (Figure 2). This is because the pyridine moieties are part of a diverse group of compounds with broad pharmacological applications ^33,34^. In addition, it is well documented that pyridine based compounds have a high probability to penetrate the CNS ^35^. Here, we focus on developing and testing five pyridine base BPA variants, which are grouped based on the structural relationship between pyridine and amide moieties: (I) one with directly attached unsubstituted pyridine moiety (pyridine-BPA), (II) and one with a separation unsubstituted pyridine moiety with one carbon (methylenepyridine-BPA), (III) with two carbons (ethylenepyridine), (IV) a benzo fused pyridine (benzopyridine-BPA, and (V) a hydroxy substituted pyridine (hydroxypyridine-BPA) (Figure 2).

**Figure 3:**
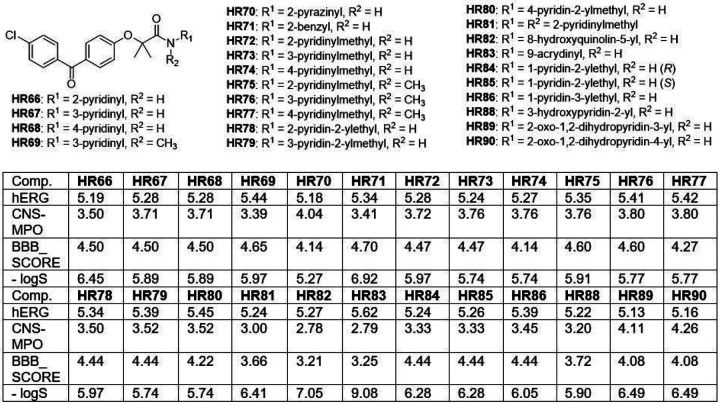
Estimated cardio potassium channel toxicity (hERG), CNS penetrability (CNS-MPO), BBB penetrability (BBB_SCORE), and water solubility (−LogS) of the selected pyridine-based **BPA** variants.

These structural variations of BPA-pyridines were designed and tested using calculated values from four algorithms: cardiotoxicity (hERG) ^36^; brain penetration capability (CNS-MPO and BBB_SCORE), and water solubility (− logS). The values for best 24 BPA-pyridines are presented in Figure 3 (for complete list of calculated values see Supplementary Materials, pages 77–138). According to our initial calculations, compounds in Figure 3 have low calculated cardiotoxicity (all predicted hERG values are below 5.5), and acceptable water solubility (estimated −logS below 7.5). In addition, CNS-MPO scores for almost all BPA-pyrimidines are over 3, which indicates probability of brain penetration above 50%. Furthermore, these compounds have the BBB_SCORE between 4 and 5, which suggests high probability for crossing the BBB.

**Figure 4:**
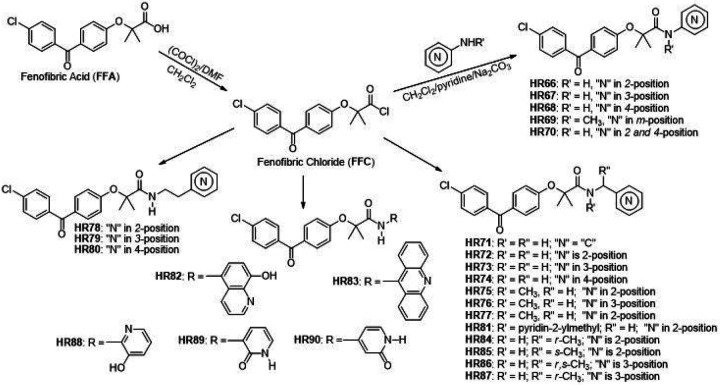
Procedure for preparation of pyridine derivatives of BPA (for more details see methods and Supplementary Materials, pages 2–56).

After determining that the proposed 24 BPA-pyridines have acceptable brain penetration ability, water solubility and low cardiotoxicity, we developed specific preparation methods foreach compound. From our previous studies, we have demonstrated that the carboxylic group of fenofibric acid (FFA) and its derivatives have exceptionally low reactivity toward nucleophilic acyl substitution. The obvious synthetic pathway for preparation of these compounds is by coupling FFA with corresponding amines ^37^. There are two major problems in performing FFA coupling with aminopyridines: (a) FFA has exceptionally low reactivity due to steric constrains generated by two methyl groups located alpha to the carbonyl group, and (b) amino groups of aminopyridines are exceptionally weak nucleophile due to amino group electron delocalization through the pyridine ring. The difficulty of performing this kind of coupling reaction was recently studied by others ^38^. However, FFA can be converted into corresponding fenofibric chloride (Figure 4), which is sufficiently reactive to aliphatic amines and activated anilines. But, coupling reactions with aminopyridines such as 4-aminopyridine is challenging at best. This is a major drawback to drug design because there are wide applications for pyridine scaffolds in medicinal chemistry ^34^. For these reasons, we have decided to explore nucleophilicity of aminopyridines through their computational data. In molecules of similar structural framework, it is possible to compare frontier molecular orbitals to determine order of nucleophilic and electrophilic reactivity ^39^. Accordingly, the molecule with highest HOMO (the highest occupied molecular orbital) are the most nucleophilic while the molecule with the lowest HOMO are the least nucleophilic via molecular comparison. We have used density functional theory to compute HOMO energies. Results provided in Supplementary Materials (page 247) indicate that aminopyridines should be less reactive (low HOMO) in comparison to aniline while aminophenol is more nucleophilic. Based on our calculations, the coupling of sterically hindered FFA through the reactive fenofibric chloride (FFC) with 4-aminophenol should give a product with a highly isolated yield. Our previous studies ^23, 40^ demonstrated that **FFC** is a viable, reactive **FFA** intermediate for amine coupling. For these reasons, we have developed a new synthetic methodology, which is shown in Figure 4, below.

There are two distinctive groups of HR compounds from the viewpoint of amine reactivity and their preparation (a) one with an aromatic heterocyclic ring directly attached to the nitrogen atom (**HR66-HR70, HR82, HR83, HR88-HR90**), and (b) one with separation of the aromatic heterocyclic ring by one or two aliphatic carbons (**HR71-HR81, HR84-HR87**). Preparation of the second group of compounds (with reactive amines) is straightforward by mixing **FFC** with corresponding amines in presence of a base. Because these amines are more nucleophilic than water and reactions are completed in several minutes at room temperature, reactions can be performed with an environmentally friendly base such as sodium carbonate in water. Isolated yields are nearly quantitative and this method (Method A) is well suited for small (milligrams) and large (hundreds of grams) scale synthesis. Also, this synthetic procedure does not require any special precautions or preparations. However, preparation of the first group, with heterocyclic aromatic ring directly attached to the amino group, requires certain precautions. Because water is a better nucleophile than these amines’, reaction must be performed in water free (dry) conditions. In addition, less nucleophilic amines are also prone to oxidation in basic conditions ^40^. Because of this, such reactions should be carried out in an oxygen free atmosphere (Method B). Under dry conditions, a pyridine solution of a corresponding aminopyridine was mixed with anhydrous sodium carbonate and kept under nitrogen atmosphere overnight. A separate dichloromethane solution of **FFC** was also prepared in dry conditions under nitrogen atmosphere. The dichloromethane solution was slowly added to cold (~0–5°C) pyridine suspension of a corresponding aminopyridine and sodium carbonate under nitrogen atmosphere. Nearly quantitative yields were obtained with reactions performed at 0 – 5°C for 3 hours, then at room temperature overnight, and finally, at 60°C for additional 3 hours. Product was isolated after solvent evaporation and water addition by simple filtration and extensive water washing. This method gave products in high yield, more the 97% purity, and did not require additional purification by either extraction or chromatography. It is also applicable to milligram and multigram preparation scales for amines with wide ranges of reactivity (Figure 4).

**Figure 5:**
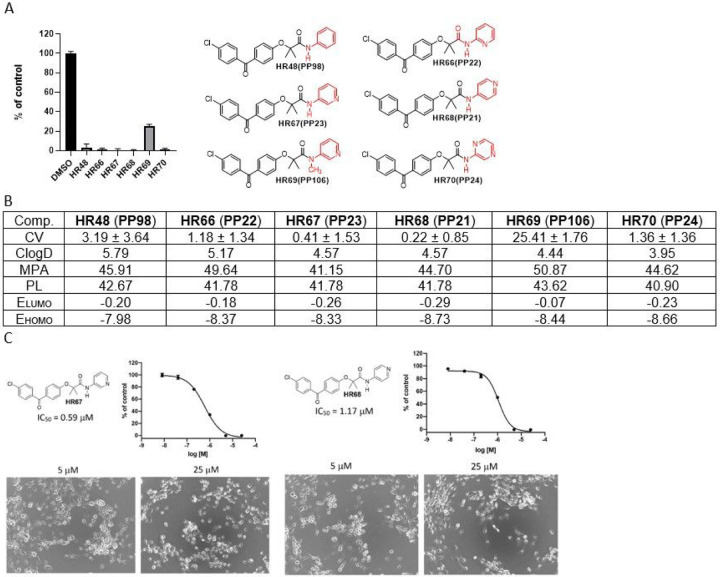
*BPA-based* *drug candidates with pyridine moiety*. ***Panel A:*** *Cell viability (MTT assay) evaluated following glioblastoma cell (LN229) exposure to 25mM of the corresponding BPA-pyrimidines. Data indicate average values with standard deviation (n=3)*. ***Panel B:*** *Tabulated values for glioblastoma-relevant parameters. CV = Cell viability (% of control) mean ± SD at 25 mM; ClogD = calculated distribution coefficient at physiological pH (lipophilicity); MPA = Minimal Projection Area (Å*^2^*); PL = Molecular Polarizability (Å*^3^*); E*_*LUMO*_ = *energy of LUMO (Lowest Unoccupied Molecular Orbital) (eV); E*_*HOMO*_ = *Energy of HOMO (Highest Occupied Molecular Orbital) (eV)*. ***Panel C:*** *IC50 graphs and pictures of the cells at 5 and 25 μM for HR67 and HR68, which are considered the most promising drug candidates in this group. Data represent average values with standard deviation (n=3).*

The first group of pyridine-BPA variants are presented in Figure 5 in which glioblastoma cell viability (CV – based on MTT assay), estimated minimal projection area (MPA), lipophilicity (ClogD), molecular polarizability (PL), as well as, energy of Lowest Unoccupied Molecular Orbital (E_LUMO_), and energy of Highest Occupied Molecular Orbital (E_HOMO_) were determined. We have included MPA based on studies showing that if the compound do not interact with cell membranes and has MPA lower than 60 Å^2^, it should be able to penetrate the CNS *via* passive diffusion ^41–43^, therefore, MPA is considered as a better parameter than molecular weight in discriminating compound ability for entering the CNS ^44^.

In the context of comparing compounds with high structural similarity, frontier orbital energies (HOMO and LUMO) can be used in determining which of the similar compounds may have a better chance for the BBB penetration ^45^. In this regard, **HR67** and **HR68** (Figure 5) both have low HOMO and LUMO energies, along with acceptable lipophilicity (ClogD), and very promising glioblastoma IC50 values (0.59 and 1.17mM, respectively), indicate that these two compound could become leading candidates for the brain tumor drug development.

**Figure 6:**
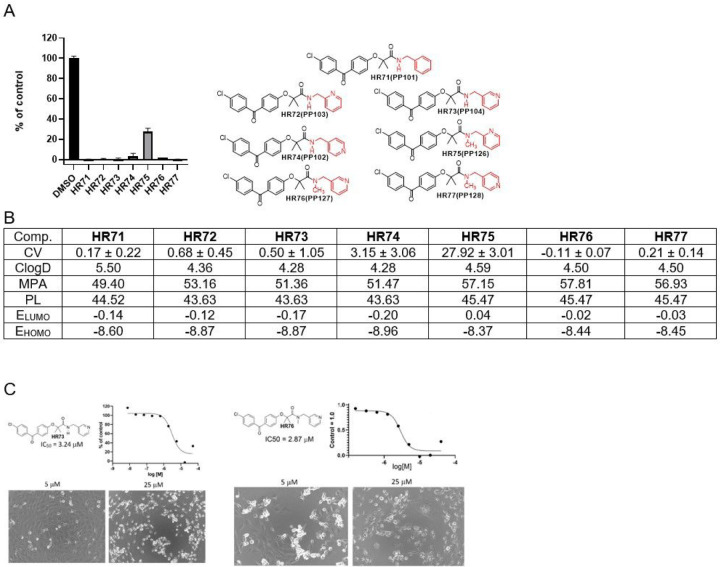
*Drug candidates with methylenepyridine moiety.* ***Panel A:*** *Cell viability (MTT assay) evaluated following glioblastoma cell (LN229) exposure to 25mM of the corresponding BPA-pyrimidines. Data indicate average values with standard deviation (n=3)*. ***Panel B:*** *Tabulated values for glioblastoma-relevant parameters. CV = Cell viability (% of control) mean ± SD at 25 mM; ClogD = calculated distribution coefficient at physiological pH (lipophilicity); MPA = Minimal Projection Area (Å*^2^*); PL = Molecular Polarizability (Å*^3^*); E*_*LUMO*_ = *energy of LUMO (Lowest Unoccupied Molecular Orbital) (eV); E*_*HOMO*_ = *Energy of HOMO (Highest Occupied Molecular Orbital) (eV). Panel C: IC50 graphs and pictures of the cells at 5 and 25 μM for HR73 and HR76, which are considered the most promising drug candidates in this group. Data represent average values with standard deviation (n=3)*.

Next group of compounds belong to methylenepyridine-BPA (Figure 6). All of these compounds show anti-glioblastoma activity at 25µM. Their estimated distributions (ClogD) are relatively high indicating high lipophilicity of the compounds. MPA values are below 60 suggesting that there is no obstacle to CNS penetration in regard to molecular size. Polarizability (PL) is between 40 and 50 indicating that these molecules can adapt to the binding area of a biomolecule through complementary polarization ^46^. Considering computed frontier orbital energies (LUMO and HOMO), HR74 should have the best binding ability. Considering experimentally determined cell viability, all **HR** compounds in Figure 6 are highly cytotoxic at 25 mM except HR75. IC50 values for the most promising compounds in this group, HR73 and HR76, are 3.24 and 2.87 mM, respectively.

**Figure 7:**
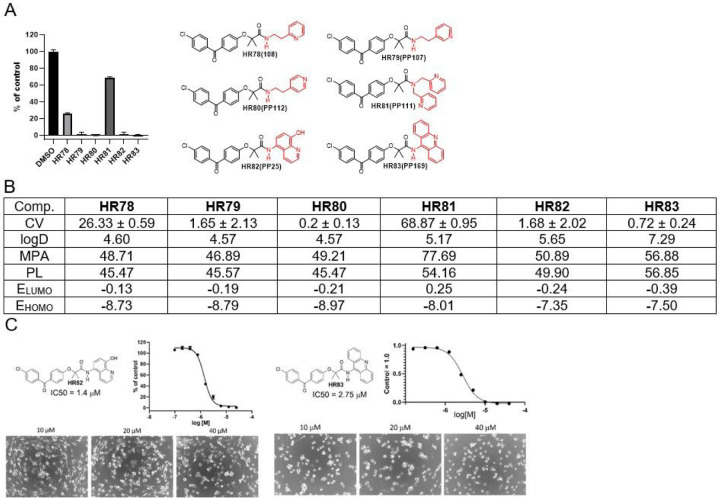
*Drug candidates with ethylenepyridine and benzopyridine moieties.* ***Panel A:*** *Cell viability (MTT assay) evaluated following glioblastoma cell (LN229) exposure to 25mM of the corresponding BPA-pyrimidines. Data indicate average values with standard deviation (n=3)*. ***Panel B:*** *Tabulated values for glioblastoma-relevant parameters. CV = Cell viability (% of control) mean ± SD at 25 mM; ClogD = calculated distribution coefficient at physiological pH (lipophilicity); MPA = Minimal Projection Area (Å*^2^*); PL = Molecular Polarizability (Å*^3^*); E*_*LUMO*_ = *energy of LUMO (Lowest Unoccupied Molecular Orbital) (eV); E*_*HOMO*_ = *Energy of HOMO (Highest Occupied Molecular Orbital) (eV)*. ***Panel C:*** *IC50 graphs and pictures of the cells at 10, 20, and 40 μM for* ***HR82*** *and* ***HR83****. Data represent average values with standard deviation (n=3)*.

Next, we asked if the compound activity would change by adding ethylene linker to ethylenepyridine-BPA or by increasing the pyridine’s molecular delocalization in benzopyridine-BPA (Figure 7). As expected, by increasing the number of carbon atoms either by adding methylene group or additional aromatic ring, lipophilicity increased noticeably (for instance, ClogD for **HR83** is 7.29). As size of the molecule increases, MPA values follow, suggesting that **HR81, HR82,** and **HR83** may have a low probabilities of penetrating the CNS. Considering the energies of both frontier molecular orbitals from three similar compounds (**HR78, HR79**, and **HR80**) **HR80** should be the most active. Indeed, obtained cell viability (CV) data for these compounds correlated with computational data and suggest that best drug candidate from this group is **HR80**. Although both benzopyridine-BPAs (HR82 and HR83) have encouraging IC50 values, 1,4 and 2.75 mM, respectively, their computed physical properties such as ClogD and MPA suggest that there is very low probability for these compounds to penetrate the CNS, further supporting HR80 as the best glioblastoma drug candidate in this group.

**Figure 8:**
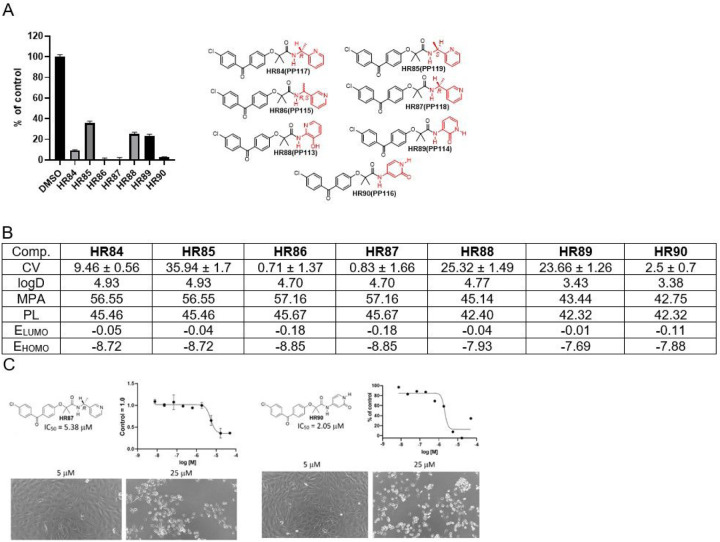
*Drug candidates with chiral methylenepyridine and hydroxypyridine moietis*. ***Panel A:*** *Cell viability (MTT assay) evaluated following glioblastoma cell (LN229) exposure to 25mM of the corresponding BPA-pyrimidines. Data indicate average values with standard deviation (n=3)*. ***Panel B:*** *Tabulated values for glioblastoma-relevant parameters. CV = Cell viability (% of control) mean ± SD at 25 mM; ClogD = calculated distribution coefficient at physiological pH (lipophilicity); MPA = Minimal Projection Area (Å*^2^*); PL = Molecular Polarizability (Å*^3^*); E*_*LUMO*_ = *energy of LUMO (Lowest Unoccupied Molecular Orbital) (eV); E*_*HOMO*_ = *Energy of HOMO (Highest Occupied Molecular Orbital) (eV)*. ***Panel C:*** *IC50 graphs and pictures of the cells at 5 and 25 μM for* ***HR87*** *and* ***HR90***. *Data represent average values with standard deviation (n=3)*.

We have also explored the importance that chirality may have on activity of these compounds (Figure 8). For instance, **HR84** and **HR85** are structural isomers of **HR78** that show moderate cytotoxicity at 25 μM (CV = 26.33). There are noticeable differences between the two stereoisomers: *R* isomer **HR84** being three times more cytotoxic than S isomer **HR85** (Figure 8). On the other hand, there is no difference between racemic **HR86** (both R&S) and optically pure R isomer **HR87**. This finding is reasonable because of the pyridine nitrogen proximity to the chiral center. One could argue that pyridine nitrogen binding is sterically diminished in **HR85** in comparison with **HR84**. Because of the position of pyridine nitrogen regarding chiral center, steric difference is diminished in **HR87**, which associates with improved cytotoxicity. The hydoxypyridine-BPAs can exist in both hydroxypyridine and amide form with the amide form being preferable by 8.97 kcal/mol according to the DFT wB97X-D/6–31G* computational method. Computational studies as well as NMR spectroscopy indicate that **HR88** is in hydroxypyridine form while **HR89** and **HR90** are in their amide form.

Of the three hydroxypyridine-BPAs, only computed data for **HR89** and **HR90** can be compared because they are in amide form while **HR88** is in the hydroxypyridine form. All computed parameters suggest that both **HR89** and **HR90** should easily penetrate the CNS. However, computed frontier orbital energies for **HR90** are lower indicating it should be more effective in penetrating the BBB. In addition, HR90-induced glioblastoma cytotoxicity is nearly 10-fold greater in comparison to HR89, making HR90 better drug candidate. In conclusion, HR87 and HR90 are the best drug candidates in this group with corresponding, glioblastoma-specific IC50 values, 5.38 and 2.05 mM, respectively (Figure 8C).

**Figure 9:**
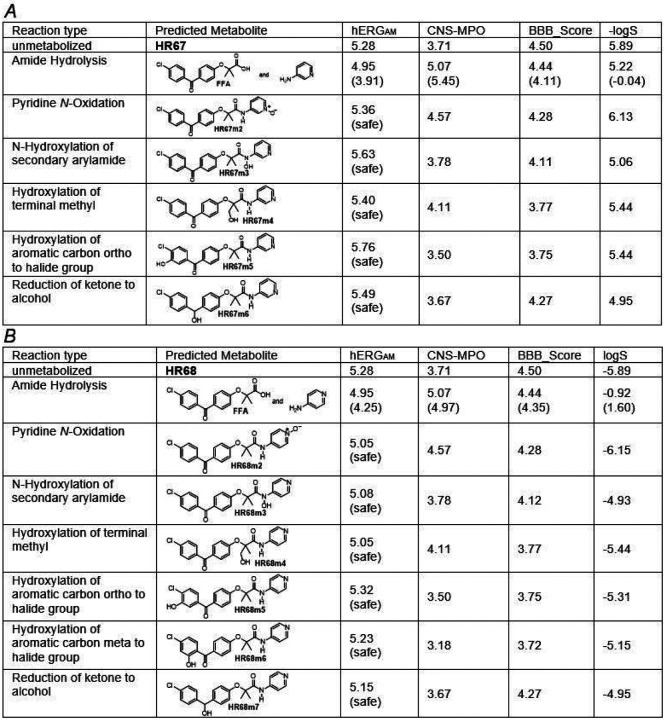
Computed properties of BioTransformer 3.0 predicted phase I metabolites of HR67 (Panel A) and HR68 (Panel B).

When pursuing new drug candidates, it is very important to envision possible metabolites and estimate their physicochemical properties, including toxicity ^47^. We used computational methods to generate metabolites of all studied compounds then evaluated their toxicity, solubility, lipophilicity and CNS penetration using the BioTransformer method ^48^. Only results for our best drug candidates, HR67 and HR68, are reported here (Figure 9). Reaction types involved in Phase I metabolism are hydrolysis, oxidation, and reduction ^49^. All predicted metabolites of HR67 and HR68 have better or comparable computed water solubility, as well as computed abilities to penetrate the CNS. Importantly, all these metabolites are predicted to be relatively safe (hERG values range 5.76 to 4.95) with the exception of amide hydrolysis, which may trigger some cardiac toxicity and should be further analyzed (hERG=3.91) (Figure 9).

**Figure 10:**
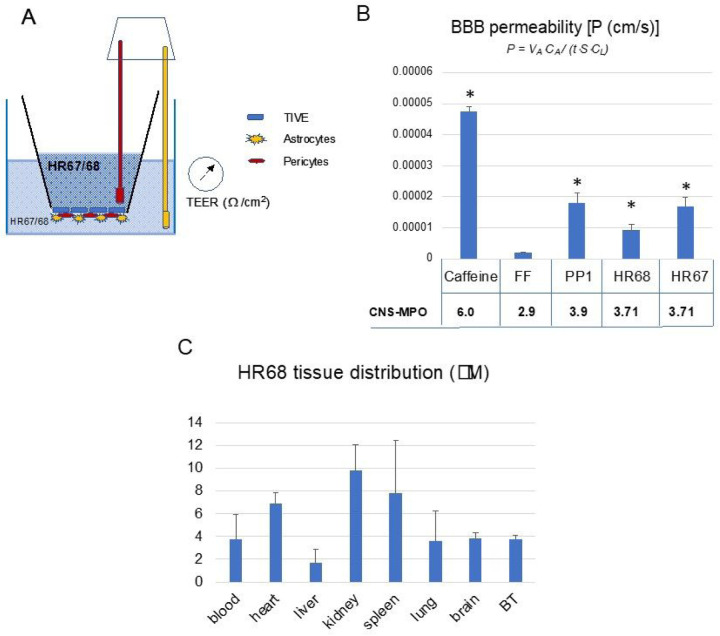
Penetration of selected PP compounds across in vitro BBB model membrane: **Panel A:** Schematic representation of a triple-coculture model of the BBB, which consists of astrocytes, pericytes and epithelial cells cultured on 24-well transwell membranes with 3mm pores. Trans-endothelial electric resistance (TEER) was measured using a EVOM^2^ meter with a STX3 electrode (World Precision Instruments). **Panel B:** BBB permeability (P) for the selected compounds was calculated using P=V_A_ ·C_A_/(t · S · C_L_) equation ^50^ and normalized by TEER coefficient. Data represent average values from 2 independent experiments in triplicates (n=6) with standard deviation SD. * indicates values significantly different from fenofibrate (negative control), and caffeine was used as positive control. **Panel C: HR68 (PP21)** tissue concentration evaluated in Foxn1 nude mice bearing intracranial glioblastoma (GBM12). Mice were treated intraperitoneally (ip) with HR68 diluted in 20% cyclodextrin at 15 mg/kg/day and the levels of HR68 in the blood, heart, liver, kidney, spleen, lung, brain and in brain tumor (BT), were evaluated by HPLC, as we previously reported ^21,24^. Data represent average values with standard deviation (n=3). **Please note that average glioblastoma IC50 for HR68 is 1.17mM, and we detected 3.7+/−0.5mM of HR68 in the brain tumor tissue**.

Our data presented in Figures 1 – 9 allowed us to perform high throughput and unbiassed selection of BPA-based compounds, which have a good chance of becoming glioblastoma drug candidates. Two pyrimidine variants of BPA, HR67 (PP23) and HR68 (PP21), were subsequently tested for their ability to penetrate artificial BBB model membranes (Figure 10A), and for tissue distribution (HR68 only), which was evaluated following intraperitoneal (ip) HR68 delivery into mice bearing patient-derived intracranial glioblastoma (Figure 10B). Although CNS-MPO scores for HR67 (3.71) and HR68 (3.71) are slightly lower compared to the CNS-MPO of our prototype drug, PP1 (CNS-MPO = 3.9) ^23,24^, these two compounds can cross the BBB model membrane (Figure 10B), and importantly, we have detected HR68 in the brain tumor tissue at concentrations over 3-fold higher than glioblastoma-specific IC50 of HR68 (1.17mM) (Figure 10C).

In conclusion, introducing pyridine moieties to the BPA scaffold improves chemo pharmacological properties of new drug candidates. In particular, water solubility and predicted CNS penetration are higher than in previously studied alkyl and phenolic derivatives of BPA ^23,51^. It was also demonstrated here that properly positioned pyridine moiety in respect to BPA increased anti-glioblastoma potency of these compounds with glioblastoma-specific IC50 values being close to 1 μM. Importantly, these specific modifications, which increased molecular flexibility and improve water solubility of the compounds, was achieved without compromising glioblastoma specific cytotoxicity. In addition, stereochemistry of the chiral center close to pyridine is important because different three dimensional orientation of pyridine nitrogen can change compound interaction with targeted biomolecules.

## Methods

### Ethics & Inclusion statement:

All intellectual, experimental and collaborative work included in this manuscript have been approved by the Louisiana Board of Ethics, and approved in accordance with the relevant guidelines and regulations by the Louisiana State University (LSU) Institutional Biosafety Committee (IBC, protocol #4351), and LSU Institutional Animal Care and Use Committee (IACUC, protocol #4966). In addition, our IACUC approved animal experiments follow 10 essential recommendations included in the ARRIVE guideline.

### Materials:

All starting materials were reagent grade and purchased from AmBeed (https://www.ambeed.com), Millipore Sigma (https://www.sigmaaldrich.com), and TCI America (https://www.tcichemicals.com). ^1^H-NMR spectra were recorded on Varian Mercury 300 and Varian Mercury 400 Plus instruments in CDCl_3_ and DMSO-d_6_, using the solvent chemical shifts as an internal standard. NMR solvents were purchased from Cambridge Isotope (https://www.isotope.com). All computed molecular descriptors were generated by ChemAxon MarvinSketch version 22.21 (https://chemaxon.com/products/marvin). All calculated values for each and every compound were performed with MarvinSketch and are included in the Supplementary materials. Frontier orbital energies, conformational studies, energy differences between various isomers and their electrostatic potential maps were calculated with wB97X-D/6–31G* Density Functional Theory (DFT) method as implemented in Spartan ‘18 v 1.1.0 (https://www.wavefun.com) and are included in the Supplementary Materials (pages 244–246). ^1^H-NMR and ^13^C-NMR spectra for all **HR** compounds generated in this study are included in Supplementary Materials. Phase I metabolites prediction was performed with BioTransformer 3.0 (https://biotransformer.ca/new) [Wishart DS, Tian S, Allen D, Oler E, Peters H, Lui VW, Gautam V, Djoumbou-Feunang Y, Greiner R, Metz TO. BioTransformer 3.0-a web server for accurately predicting metabolic transformation products. ^52^

### **Method A**.

(General method applicable for preparation of all **HR66-HR90** compounds in large scale without extraction or crystallization). ***Preparation of 2-[4-(4-chlorobenzoyl)phenoxy]-2-methyl-N-(pyridin-3-yl)propenamide (HR67)***. Dry pyridine suspension of 3-aminopyridine (14.2 g; 0.15 mol) and anhydrous sodium carbonate (42.4 g; 0.4 mol) was sonicated for thirty minutes and left at room temperature in a closed system overnight under nitrogen atmosphere to ensure that the pyridine suspension remained dry. Separately, fenofibric chloride (FFC) was prepared as follows: Dichloromethane (500 ml) suspension of fenofibric acid (47.4 g; 0.15 mol), oxalyl chloride (25.7 ml; 38.1 g; 0.3 mol), and DMF (few drops) were stirred at room temperature overnight. After approximately 1.5 hours, the reaction mixture became light brown. The majority of solvent was removed by distillation at atmospheric pressure and the remaining solvent was removed under Argon flow at room temperature. The resulting solid material was dissolved in dichloromethane (150 ml),under nitrogen atmosphere and with slow stirring, was added to the previously prepared pyridine suspension of 3-aminopyridine and sodium carbonate cooled with ice-water. The resulting suspension was stirred at 0–5°C for 3 hours, then at room temperature overnight, and was followed by stirring at 60°C for additional 3 hours. The resulting solvent was removed under reduced pressure to separate the solid residue. This residue was mixed with water (1 L) and stirred *via* sonication for 4 hours. The insoluble white crystalline product was separated by filtration, extensively washed with water (20 ×100 ml), and dried at 60°C, under vacuum. The isolated yield was 90% (53.3 g). ^1^H-NMR (DMSO-d_6_, 400 MHz) δ 10.31 (1H, s), 8.83 (1H, s), 8.26 (1H, d, *J* = 4.4 Hz), 8.06 (1H, d, *J* = 8.4 Hz), 7.72 (2H, d, *J* = 8.4 Hz), 7.67 (2H, d, *J* = Hz), 7.54 (2H, d, *J* = 8.4 Hz), 7.31 (1H, d of d, *J*_1_ = 8.4 Hz, *J*_2_ = 4.4 Hz), 7.04 (2H, d, *J* = 8.8 Hz), and 1.63 (6H, s) ppm. ^13^C-NMR (DMSO-d_6_, 100 MHZ) δ 193.7, 172.9, 159.4, 145.2, 142.6, 137.6, 136.6, 135.6, 132.3, 131.6, 130.6, 129.0, 128.0, 123.9, 118.8, 81.5, and 25.2 ppm.

### **Method B**.

General preparation of **HR71-HR81** and **HR84-HR87** in large scale with sodium carbonate in water as base. ***Preparation of 2-(4-(4-chlorobenzoyl)phenoxy)-2-methyl-N-(2-(pyridin-4-yl)ethyl)propenamide (HR80)***. Fenofibric chloride (**FFC**) (0.3 mol) was prepared by the following procedure described in Method A from fenofibric acid (95.6 g; 0.3 mol) and oxalyl chloride (63.5g; 43 ml) in dichloromethane (1L). Prepared FFC (0.3 mmol) was slowly added dropwise over a period of 45 minutes at ice-water bath temperature into a magnetically stirred mixture of sodium carbonate (106 g; 1 mol) in water (500 ml) and 1-(pyridine-4-yl) ethanamine (24.4 g; 0.2 mmol) in tetrahydrofuran (500 ml). After addition was complete, the resulting reaction mixture was stirred at room temperature overnight. The reaction mixture volume was reduced by 75% via solvent evaporation under air flow (produced by air pump). The resulting white suspension was mixed with water (500 ml) and the insoluble product was separated by filtration, washed with water (10×50 ml), and dried at 50°C overnight. The isolated yield was 97% (82 g) of pure product. The filtrate was acidified with hydrochloric acid to pH = 2. The resulting white solid precipitate was separated by filtration, washed with water (10×20 ml) and dried at 50°C overnight to give 34.2 g (95% recovery) of fenofibric acid. ^1^H-NMR (DMSO-d_6_, 400 MHz) δ 8.36 (2H, d, *J* = 4.8 Hz), 8.21 (1H, t, *J* = 5.2 Hz), 7.66 (6H, m), 7.13 (2H, d, *J* = 5.2 Hz), 6.86 (2H, d, *J* = 8.0 Hz), 3.85 (2H, m), 2.73 (2H, t, *J* = 7.2 Hz), and 1.45 (6H, s) ppm. ^13^C-NMR (DMSO-d_6_, 400 MHz) δ 193.7, 173.1, 159.6, 149.7, 148.7, 137.5, 136.7, 132.1, 131.6, 130.2, 129.1, 124.6, 118.7, 81.1, 39.4, 34.3, and 25.5 ppm.

### Method C.

Small scale preparation applicable to all **HR66-HR90*. Preparation of 2-(4-(4-chlorobenzoyl)phenoxy)-2-methyl-N-(2-oxo-1,2-dihydropyridin-3-yl)propanamide (HR89)***. Freshly prepared dichloromethane (5 ml) solution of fenofibric acid chloride was made from fenofibric acid (80 mg; 0.25 mmol) and oxalyl chloride (1 mmol) as described above. These were added, under a nitrogen atmosphere while stirring, to a pyridine (10 ml) - tetrahydrofuran (10 ml)-sodium carbonate (212 mg; 2 mmol) of 3-aminopyridin-2(1*H*)-one (27.5 mg; 0.25 mmol) solution. The resulting mixture was stirred at room temperature in the nitrogen atmosphere for 3 hours, followed by stirring under the nitrogen atmosphere at 60°C for an additional 3 hours. After cooling to room temperature, the solvent was evaporated under air flow (produced by air pump) at room temperature yielding a solid residue. This solid residue was mixed with dichloromethane (30 ml) and? water (100 ml). The water layer was discarded, and the organic layer was washed with water (3×100 ml), 5% sodium carbonate (3×100 ml), and dried over anhydrous sodium carbonate. The drying material was separated by filtration. The volume of the filtrate was reduced to ~ 2 ml, then hexanes (~10 ml) were added. The resulting solution was left uncovered at room temperature, allowing the solvent to slowly evaporate. The resulting white crystalline product was separated by filtration, washed with hexane (3×3ml) and air-dried overnight. Isolated yield=93% (95 mg). ^1^H-NMR (DMSO-d_6_, 400 MHz) δ 12.08 (1H, s), 9.29 (1H, s), 8.24 (1H, d of d, *J*_1_ = 7.2 Hz, *J*_2_ = 1.6 Hz), 7.73 (2H, d, *J* = 8.8 Hz), 7.71 (2H, d, *J* = 8.8 Hz), 7.59 (2H, d, *J* = 8.4 Hz), 7.12 (1H, m), 7.10 (2H, d, *J* = 8.8 Hz), 6.26 (1H, t, *J* = 7.2 Hz), and 1.58 (6H, s) ppm. ^13^C-NMR (DMSO-d_6_, 100 MHz) δ 193.8, 172.4, 158.5, 157.6, 137.7, 136.4, 132.3, 131.7, 131.6, 129.1, 128.7, 128.6, 123.1, 120.3, 105.9, 82.3, and 25.2 ppm.

### Cell culture and viability assays.

Human glioblastoma LN-229 cells (ATCC CRL-2611) were maintained as a semi-confluent monolayer culture in DMEM with 1 g/L glucose, sodium pyruvate and L-glutamine (Corning), supplemented with 10% heat-inactivated FBS (Gibco) and P/S (50 units/mL of penicillin and 50 μg/mL of streptomycin) at 37°C in a 5% CO_2_ atmosphere. Prior to treatment with HR compounds, cells were plated in 96-well plates (BD Falcon) at an initial density of 2 × 10^4^ cells/cm^2^. Twenty-four hours after plating, stock solutions of HR compounds were prepared in DMSO, diluted in cell culture medium and added to previously plated cells in triplicate for every experimental condition (final concentration 25 μM). DMSO (0.5%) was used as vehicle control. After 72h incubation, an MTT assay was performed to measure cell metabolic activity (surrogate for cell viability). Following a 1.5 h incubation with 0.5 mg/ml MTT in serum free low glucose DMEM, the resulting formazan crystals were dissolved in 5mM HCl in isopropanol and the absorbance read at 540 nm. Data represent mean values expressed as the percentage of vehicle control ± SD. Phase contrast images of treated cells were taken 72 hours after treatment with HR compounds using a BZ-X800 fluorescence microscope (Keyence) equipped with a 20x objective. The drug dose resulting in 50% inhibition of cell metabolic activity (surrogate for cell viability) was measured using MTT assay, at 72-hour time point, and half maximal inhibitory concentration (IC50) was calculated using GraphPad Prism 8.

### *In vitro* model of the Blood Brain Barrier (BBB).

The BBB was re-created *in vitro* using a modified protocol provided by Stone *et al* ^53^. Briefly, 24-well transwell inserts (Falcon, catalog number 353096) were coated with 10mg/cm^2^ of Collagen Type IV (Sigma) for 24 hrs at 4°C. Inserts were washed with sterile water and air-dried for 2 hrs. Next, the inserts were coated with 2mg/cm^2^ poly-L-lysine (ScienCell) for 1 hr at 37°C, then washed twice with sterile H_2_0 and air-dried for 2 hrs. Primary human astrocytes (1.5×10^5^) and 3×10^4^ primary human pericytes (both ScienCell) were resuspended in 25ml of astrocyte medium and pericyte medium (ScienCell), respectively, then combined in a 1:1 ratio for 50ml total volume. Dried, coated inserts were turned upside down such that the basolateral surface was exposed at the top, and 50ml of the cell mixture was added to the membrane, covered with the plate lid, and incubated for 2 hrs at 37°C to allow cell adhesion. Any medium remaining on top of the membrane was carefully removed before returning inserts to their upright position with the apical surface facing upward, as they were placed in a 24-well plate containing 500ml *per* well of astrocyte/pericyte medium (1:1). An additional 300 mL of medium was added to the apical compartment. Four days after plating, the apical compartment medium was removed, and 3.75×10^4^ of telomerase-immortalized vain endothelial cells (TIVE; provided by Dr. Rolf Renne) in 50ml of TIVE medium ^54^ were added and incubated for 5 hrs at 37°C to allow cell attachment, followed by the addition of an extra 250ml of TIVE medium. Half the volume of the corresponding media in the lower and upper compartment was replaced with fresh media every third day. Ten days after initial plating, trans-endothelial electric resistance (TEER) was measured using a EVOM^2^ meter with a STX3 electrode (World Precision Instruments). The ability of selected HR compounds to pass through the *in vitro* BBB was tested using inserts with effectively reconstructed BBB as confirmed by TEER values ^53,55^.

### High Performance Liquid Chromatography (HPLC)-based detection of selected HR compounds:

Following TEER measurement, the medium from the apical compartment (insert) of the *in vitro* BBB model (Fig. 10A) was replaced with 350mL of fresh TIVE medium containing corresponding compounds [HR67 (PP23), HR68 (PP21), both used at 25mM. In addition, 25mM fenofibrate (FF), which does not cross the BBB ^22^,was used as a negative control, and 50mM caffeine was used as a positive control ^56^. Plates containing the inserts were returned to the incubator (37°C, 5% CO_2_), and after 3 hrs of incubation, conditioned media from the well and insert (Fig. 10A) were collected. The aliquots (100μl) of the collected samples were subsequently mixed with 100μl of 100% acetonitrile, centrifuged (16.000 rpm at 4°C for 10 min) and supernatants collected for HPLC -based detection of HR67 and HR68.

HPLC analyses were performed using an UltiMate 3000 system (Thermo Scientific) equipped with an analytical YMCbasic, 3µm, 150 × 4.6 mm column (octyl silane C8; YMC America, Inc.). Isocratic elution of the compounds was performed using a mobile phase composed of solvent A (50 mM acetic acid in dH_2_O) and solvent B (acetonitrile) mixed at predetermined ratios for each compound (Table 1). All separations were carried out with 5μl sample volume at a flow rate of 1 ml/min, at 25°C. The concentration of each compound was calculated using serial dilutions of the known concentration of the compound separated at the same run with experimental and control samples. After separation, integrated areas under the peak were used to prepare calibration curves and to determine concentration of the compounds.

### Intracranial Glioblastoma and Tissue Extraction:

Foxn1nu female immunodeficient mice at 6 to 8 weeks of age were used in this study (both male and female are similarly affected by glioblastoma). The mice were inoculated with patient-derived glioblastoma, GBM12-TMZ-resistant, which stably express luciferase reporter ^24,57^, were kindly provided by Dr. Sarkaria (Cleveland Clinic, Brain Tumor National Resource) and were cultured and propagated according to the recommended protocols ^57^. The cells were injected into the striatum region using 5ml of PBS containing 1×10^5^ of the tumor cells guided by the stereotactic approach [1.5 mm posterior to Bregma; 1.5 mm lateral to Sagital suture; 3 mm down from surface] as reported in our previous study ^24^. The treatment started when the intracranial tumors were well-established (evaluated by the Optical Image System for small animals (Xenogen IVIS CT). HR68 injection solution was prepared from the 50mM DMSO stock solution diluted in 20% cyclodextrin (2-Hydroxypropyl-β-cyclodextrin) in sterile PBS and delivered intraperitoneally (*ip*) at 15 mg/kg. Blood, liver, kidneys, spleen, heart, brain and brain tumor were subsequently collected, solid tissues were washed from blood in PBS, and placed on ice for an immediate sample preparation for HPLC analysis (see above). Tissues were prepared for HPLC by mixing 150 μl of sample tissue (~120 mg) that had been mixed with 3 volumes of Methanol : H_2_O mix (4:1), well-blended using TissueRuptor II (Qiagen), and centrifuged at 15,000g for 10 minutes at 4^o^C. Supernatants were collected in 1.5 ml Eppendorf tubes and incubated at 95°C for 3 min. Following flash cool on ice, samples were centrifuged again at 15,000g for 10 minutes at 4°C and supernatants were used for HPLC-based measurement.

### Statistical analysis.

The data were analyzed with a homoscedastic Student t test. Differences between control and experimental groups were considered significant at P values of £ 0.05.

## DATA AVAILABILITY STATEMENT:

The datasets used and/or analyzed during the current study are available from the corresponding author on reasonable request.

## Figures and Tables

**Table1.** Details of the HPLC method for selected HR compounds.

Compound	Method length [min]	Concentration solvent B [%]	Detection wavelength [nm]	Retention time [min]
Caffeine	5	25	272	2.54
Fenofibrate	10	70	288	5.84
HR67	10	60	268	4.45
HR68	10	60	266	4.45
